# Vector-Free and Transgene-Free Human iPS Cells Differentiate into Functional Neurons and Enhance Functional Recovery after Ischemic Stroke in Mice

**DOI:** 10.1371/journal.pone.0064160

**Published:** 2013-05-23

**Authors:** Osama Mohamad, Danielle Drury-Stewart, Mingke Song, Ben Faulkner, Dongdong Chen, Shan Ping Yu, Ling Wei

**Affiliations:** 1 Department of Anesthesiology, Emory University School of Medicine, Atlanta, Georgia, United States of America; 2 Department of Biomedical Engineering, Georgia Tech Institute, Atlanta, Georgia, United States of America; National Institute on Aging Intramural Research Program, United States of America

## Abstract

Stroke is a leading cause of human death and disability in the adult population in the United States and around the world. While stroke treatment is limited, stem cell transplantation has emerged as a promising regenerative therapy to replace or repair damaged tissues and enhance functional recovery after stroke. Recently, the creation of induced pluripotent stem (iPS) cells through reprogramming of somatic cells has revolutionized cell therapy by providing an unlimited source of autologous cells for transplantation. In addition, the creation of vector-free and transgene-free human iPS (hiPS) cells provides a new generation of stem cells with a reduced risk of tumor formation that was associated with the random integration of viral vectors seen with previous techniques. However, the potential use of these cells in the treatment of ischemic stroke has not been explored. In the present investigation, we examined the neuronal differentiation of vector-free and transgene-free hiPS cells and the transplantation of hiPS cell-derived neural progenitor cells (hiPS-NPCs) in an ischemic stroke model in mice. Vector-free hiPS cells were maintained in feeder-free and serum-free conditions and differentiated into functional neurons *in vitro* using a newly developed differentiation protocol. Twenty eight days after transplantation in stroke mice, hiPS-NPCs showed mature neuronal markers *in vivo*. No tumor formation was seen up to 12 months after transplantation. Transplantation of hiPS-NPCs restored neurovascular coupling, increased trophic support and promoted behavioral recovery after stroke. These data suggest that using vector-free and transgene-free hiPS cells in stem cell therapy are safe and efficacious in enhancing recovery after focal ischemic stroke in mice.

## Introduction

Stroke is a leading cause of human death and disability in the adult population in the United States and many countries around the world. In the United States alone, there is average one stroke victim every 40 seconds and about 795,000 individuals experience a stroke every year [1]. Despite the substantial health and economic burden of stroke, clinical trials have failed to establish the therapeutic benefit of hundreds of candidate drugs that showed various beneficial effects in animal models [2], [3]. Alternatively, cell-based therapies using a variety of embryonic and adult stem cells are currently being investigated as potential regenerative and/or protective treatments for ischemic stroke.

Stem cell transplantation may repair damaged tissues and enhance endogenous repair mechanisms after ischemic stroke [4]. Previous studies from our lab and others have shown that transplantation of embryonic stem (ES) cells improves functional recovery after ischemic stroke [5], [6]. On the other hand, the derivation and application of human ES cells raise ethical concerns that hinder basic and clinical research [7]. Recently, a cocktail of transcription factors has been shown to reprogram mouse [8] and human [9], [10] fibroblasts to pluripotent stem cells that have the differentiation potential of becoming all three germ layer cells. These induced pluripotent stem (iPS) cells are genetically, epigenetically [8], [11], and morphologically similar (but not the same) to ES cells, with comparable differentiation capacities [12], [13]. Moreover, many protocols have been established to differentiate iPS cells to neurons and use them to treat or model neurodegenerative diseases [14]–[21]. iPS cell derivation has substantially developed over the past 5 years [22]–[28]. Initially, iPS cells were produced by lenti-viral constructs carrying transcription factors that include c-myc. However, both c-myc and the lenti-viral DNA contribute to a potential cancerous transformation of iPS cells after transplantation. More recently, iPS cells were produced by using non-integrating episomal vectors which circumvent the continuous presence of both lentiviral DNA or c-myc [29]. Despite the great potential of iPS cells, the use of vector-free hiPS cells as a transplantation therapy for ischemic stroke has not been explored yet.

In this paper, we report the use of vector-free hiPS-NPCs in animal models of ischemic stroke. We demonstrate successful hiPS cell cultures in feeder- and serum-free conditions and high-efficiency *in vitro* differentiation to functional neurons. We also demonstrate *in vivo* neuronal differentiation and enhanced functional recovery and trophic support after transplantation in stroke animals. In summary, vector-free hiPS cells appear to be a plausible alternative to human ES cells in cell based therapies after ischemic stroke.

## Materials and Methods

### Human iPS Cells and Culture Condition

Vector-free transgene-free hiPS cells (iPS-DF19-9/7T) were purchased from the WISC stem cell bank (WiCell Research Institute, Madison, WI). The cells used for differentiation and transplantation were no older than passages 30–40. hiPS cells were maintained in feeder- and serum-free media (mTeSR1, Stem Cell Technologies, Vancouver, BC, Canada) on hES-qualified Matrigel (BD Biosciences, Sparks, MD) [30]. mTeSR1 media was changed every day and cells were passaged using dispase every 5–7 days after manual removal of differentiated colonies. For more information on the maintenance of hiPS cells with mTeSR1, please refer to the guidelines published by Stem Cell Technologies.

### Quantitative Real-time Polymerase Chain Reaction (qRT-PCR)

Total RNA was extracted from cell cultures (hiPS and hiPS-NPCs) using the TRIzol reagent (Invitrogen Inc, Carlsbad, CA). Reverse transcription was performed with 1 µg total RNA using the High Capacity cDNA Reverse Transcription kit (Applied Biosystems, CA, USA). SYBR green qRT-PCR was used to assess the relative levels of our target genes using the Applied Biosystems StepOnePlus machine. The primers used are similar to those used in [31]. Fold change was calculated by the delta (delta Ct) method using GAPDH amplification as the internal control.

### Neural Induction Protocol

To obtain neural precursors, we used a modification of the adherent differentiation protocol described previously [14], [32]. Cells were dissociated using accutase (Invitrogen, Carlsbad, CA) for 15 min and then plated on Matrigel (BD Biosciences) coated plates at a density of 18,000–20,000 cells/cm^2^ in mouse embryonic fibroblast (MEF) conditioned medium [plus 10 ng/ml basic fibroblast growth factor (human recombinant bFGF, R&D, Minneapolis, MN) and 10 µM ROCK inhibitor (Y27632, Sigma, St. Louis, MO)]. When cells reached confluence (3–5 days later), the medium was changed to KSR medium (Knockout DMEM, 15% knockout serum replacement, 1× L-glutamine, 1× non-essential amino acids, 50 mM β-mercaptoethanol) with the addition of 3 µM dorsomorphin (Tocris, Ellisville, MO) and 10 µM SB431542 (Stemgent, Cambridge, MA). This is considered day 0 of the neural induction protocol. Cells were grown in this media for five days, with daily media changes. On day 5, media was changed to a 1∶4 mixture of N2:KSR media (N2 media has DMEM/F12, N2 supplement, 1× L-glutamine, penicillin/streptomycin; Invitrogen) without the TGF-β inhibitor (SB431542). On days 7 and 9, the media was changed to 1∶1 and 4∶1 N2:KSR media, respectively. On day 11 of the neural induction protocol, neural precursors were collected for Western blotting, fixed for staining or dissociated with accutase for transplantation or for terminal differentiation. For terminal neuronal differentiation, neural precursors were dissociated into a single cell suspension using accutase and then filtered through a 200 µm mesh. 100,000–150,000 cells were plated on Matrigel in a 1∶1 mixture of N2 and B27 medium (B27 media has Neurobasal media, B27 supplement, 1× L-glutamine, penicillin/streptomycin; Invitrogen) with 10 ng/ml bFGF. Media was changed every third day for 4 weeks. Four weeks later, cells were clamped for electrophysiological recording and then fixed with 4% PFA for staining.

### Immunocytochemistry

For immunocytochemictry, cells were fixed with 4% paraformaldehyde, post-fixed with a 2∶1 mixture of ethanol:acetic acid, permeabalized with 0.2% Triton-X-100, and blocked with 1% fish gelatin. Primary antibodies [Nanog (1∶400), OCT4 (1∶400), SOX2 (1∶400), β-III-Tubulin (1∶200) (Cell Signaling, Danvers, MA); Tuj-1 (1∶400), PAX6 (1∶100) (Covance, Princeton, NJ); Nestin (1∶200), NeuN (1∶400), Neurofilament (1∶400), MAP2 (1∶400) (Millipore; Synapsin 1 (1∶400) (Calbiochem)] were applied overnight at 4°C and Alexafluor or Cy-3 conjugated secondary anti-rabbit or anti-mouse antibodies (1∶300) were used. Hoechst-33342 (Molecular Probes, Invitrogen) was used to counter-stain cell nuclei. All images were taken using an upright Olympus fluorescence microscope.

### Western Blot

Western blot analysis was performed to analyze protein expression in hiPS-NPCs and from brain tissue after transplantation following previous procedures [33]. In brief, cells were scraped from dishes using lysis buffer. Brain tissue was also lysed in lysis buffer containing 25 mM Tris–HCl pH 7.4, 150 mM NaCl, 5 mM EDTA, 0.1% SDS, 2 mM sodium orthovanadate, 100 mM NaF, 1% triton, leupeptin, aprotinin, and pepstatin with continuous manual homogenization. Lysate was then spun at 13,000 rpm for 15 minutes and supernatant was collected. Protein concentration was determined using BCA protein assay (Pierce, Rockford, IL). Equal amounts of protein were resolved on SDS-PAGE using gradient gels (6–18%) and gels were blotted onto PVDF membranes (Amersham, Buckinghamshire, UK), blocked with 5% BSA in TBST buffer (20 mM Tris, 137 mM NaCl and 0.1% tween) and incubated overnight with primary antibodies against erythropoietin EPO (1∶50, Santa Cruz), EPO-receptor (1∶50, Santa Cruz), vascular endothelial growth factor VEGF (1∶100, Novus Biologicals), Flt-1 or VEGF-receptor 1 (1∶50, Santa Cruz), VEGF-receptor 3 (1∶500, Chemicon), brain-derived neurotrophic factor BDNF (1∶50, Santa Cruz), CXCR4 (1∶500, R&D), Tie-1 and Tie-2 (1∶50, Santa Cruz), Angiopoietin-1 (1∶500, Abcam), and Angiopoietin-3 (1∶400, Oricogene). After 3 washes with TBST, blots were incubated with HRP-conjugated secondary antibodies (anti-rabbit or anti-mouse, 1∶2000, Bio-Rad, CA) in 5% BSA for 1 hr. Blots were developed using Pierce ECL Western Blotting Substrate (Thermo Scientific, IL). The level of protein expression was normalized to β-actin controls.

### Electrophysiology

Whole-cell patch clamp recording was obtained from hiPS cell-derived neurons 4 weeks (28 days) after terminal differentiation using an EPC9 amplifier (HEKA Elektronik, Lambrecht, Germany) at 21–23°C. The external solution contained 135 mM NaCl, 5 mM KCl, 2 mM MgCl_2_, 1 mM CaCl_2_, 10 mM HEPES, and 10 mM glucose at a pH of 7.4. Internal solution consisted of 120 mM KCl, 2 mM MgCl_2_ 2, 1 mM CaCl_2_, 2 mM Na_2_ATP, 10 mM EGTA, and 10 mM HEPES at a pH of 7.2. Recording electrodes pulled from borosilicate glass pipettes (Sutter Instrument, USA) had a tip resistance between 5 and 7 MΩ when filled with the internal solution. Series resistance was compensated by 75–85%. Linear leak and residual capacitance currents were subtracted on-line using a P/6 protocol. Action potentials were recorded under current-clamp mode using Pulse software (HEKA Elektronik). Data were filtered at 3 KHz and digitized at sampling rates of 20 KHz.

### Transient Focal Ischemia Animal Model

All experimental and surgical procedures were approved by the Institutional Animal Care and Use Committee (IACUC) at Emory University. Middle cerebral artery occlusion (MCAo) was performed according to a modified version of [34]. In brief, 8–10 weeks old C57BL6 mice (National Cancer Institute) were anesthetized using an intraperitoneal (IP) injection of 4% chloral hydrate. The right middle cerebral artery (MCA) was permanently ligated by a 10-0 suture (Surgical Specialties Co., Reading, PA) accompanied by a bilateral 7-min ligation of the common carotid arteries (CCA). During CCA occlusion, barrel cortex blood flow was reduced to less than 20% as measured by laser Doppler scanning (Fig. S1A) (p<0.05, Student’s *t-test*). We used laser Doppler blood flow imaging to determine local cerebral blood flow in the control and the transplantation groups from six adjacent areas around the medial border of the infarct area before and during stroke and 3, 7, and 14 days post-transplantation as described in [35]. Stroke was visualized using the 2,3,5-triphenyltetrazolium chloride (TTC) staining procedure detailed in [36]. Body temperature was monitored during surgery and maintained at 37.0°C using a temperature control unit and heating pads. Animals were euthanized by decapitation at different time points after ischemic stroke. Brains were immediately removed, mounted in optimal cutting temperature compound (Sakura Finetek USA, Inc., Torrance, CA) and stored at −80°C for further processing.

### Cell Transplantation after Ischemic Stroke

Seven days after stroke, mice were anesthetized for the transplantation of hiPS-NPCs or media vehicle. On day 11 after neural induction, neural precursors were treated with Hoechst-33342 (1∶10,000) [37], [38] for one hour, dissociated into a single cell suspension using accutase, filtered through a 200 µm mesh and then re-suspended in N2 medium. Each animal received an injection of 4 µl transplantation solution (100,000 cells/µl or media vehicle) at 2 different sites (the ischemic core and the penumbra region). The solution was injected using a 5 µl Hamilton syringe (Hamilton Company). Cells were injected very slowly (total time of injection 10 min at each location) and the needle was allowed to stay for 5 min after the injection to allow for diffusion of the transplanted cells. The mortality/failure rate from the surgery and transplantation procedures is less than 15%. All surviving animals were tested according to experimental plan until sacrifice.

### Histological and Immunohistochemical Assessments and Cell Counting

Animals were sacrificed 28 days after transplantation and their brains immediately frozen on dry ice. Brain coronal sections were cut at 10 µm thickness using a cryostat (Leica CM 1950). Staining for NeuN and Collagen IV was performed following previously described protocols [39], [40]. In brief, sections were dried on a slide warmer for 30 min and fixed with 10% buffered formalin for 10 min. Brain sections were washed with −20°C pre-cooled ethanol:acetic acid (2∶1) solution for 10 min and finally permeabilized with 0.2% Triton-X 100 (in PBS) for 5 min. Sections were then blocked with 1% donkey serum (Sigma) in PBS for 1 hr at room temperature, and incubated with the primary antibodies collagen IV (CoIV; 1∶400; Millipore, CA, USA) and NeuN (1∶400; Millipore, MA, USA) overnight at 4°C. Slides were incubated with anti-mouse and anti-goat secondary antibodies for 1 hr at room temperature. Vectashield mounting media for fluorescence (Vector Laboratory, Burlingame, CA) was used to cover-slip slides for microscopy and imaging analysis. Cell count was performed following a modification of the principles of design based stereology. Systematic random sampling was employed to ensure accurate and non-redundant cell counting [41]. Every section under analysis was at least 100 µm away from the next. For each animal, six 10-µm thick sections spanning the entire region of interest were randomly selected for cell counting. Counting was performed on 6 randomly selected non over-lapping fields per section. Sections from different animals represent the same area in the anterior-posterior direction. Nissl staining was performed by first fixing the sections in a 1∶1 mixture of formalin and acetic acid for 10 min. Sections were then placed in a working solution of Cresyl violet, acetic acid and sodium acetate for 20 min, rinsed with 70% ethanol and finally dried overnight.

### Terminal Deoxynucleotidyl Transferase dUTP Nick End Labeling (TUNEL) Staining of Cell Death

TUNEL staining was performed using a commercial kit (DeadEnd™ Fluorometric TUNEL system, Promega, Madison, WI, USA) to label dying and dead cells in the brain 48 hrs after transplantation. The instructions were followed as dictated in the instructions manual. In brief, brain sections were placed in equilibration buffer and incubated with nucleotide mix and rTdT enzyme at 37°C for 1 hr. The reaction was stopped with 2× SSC.

### Adhesive Removal Test

The adhesive removal test was performed according to the procedure described before [42]. In brief, a piece of adhesive tape was placed on each (right and left) forepaw and the time-to-contact (latency) and the time-to-remove (removal) the tape was recorded. Animals were trained for three days before stroke induction to get a basal level of performance. The test was performed before stroke (training), just before transplantation, and 7, 14, 21 and 28 days after transplantation. The final result (fold change compared to baseline) is the ratio of the time-to-contact (or time-to-remove) at each time point to the time-to-contact (or time-to-remove) immediately after training and is the average of 3 trials separated by at least 15 min at each time point.

### Intrinsic Optical Signal (IOS) Imaging

IOS imaging was used to assess the local neuronal activity [34]. IOS imaging is a method to measure changes in neurovascular coupling after stroke. The detected signals indicate an increase in blood flow in the activated barrel corresponding to stimulations of specific whiskers. IOS imaging was performed on normal sham control, stroke plus media injection, and stroke plus hiPS-NPCs transplanted mice with or without whisker stimulation. Media control and hiPS-NPC transplantation (30 days after transplantation) animals were anesthetized and the exposed cortex was rinsed with sterile buffered saline at 37°C and cover-slipped before imaging under green light (570 nm) with a CCD camera. The green light is transmitted to the penumbra region through the same cranial window used for stroke induction. We detected the signal reflected by blood hemoglobin after absorbing the green light. The resulting images were processed using ImageJ (NIH). Five animals were imaged in each group.

### Statistical Analysis

All results are expressed as mean ± S.E.M. Statistical comparisons were made with Student’s *t-test* or two-way analysis of variance (ANOVA) with Bonferroni’s *post-hoc* analysis to identify significant differences. *P*<0.05 was considered significant for all comparisons. All experiments and statistical analyses were performed by researchers who were blinded to the nature of the experimental groups.

## Results

### Characterization of hiPS Cells Cultured in Serum-free and Feeder-free Media

**Figure 1:**
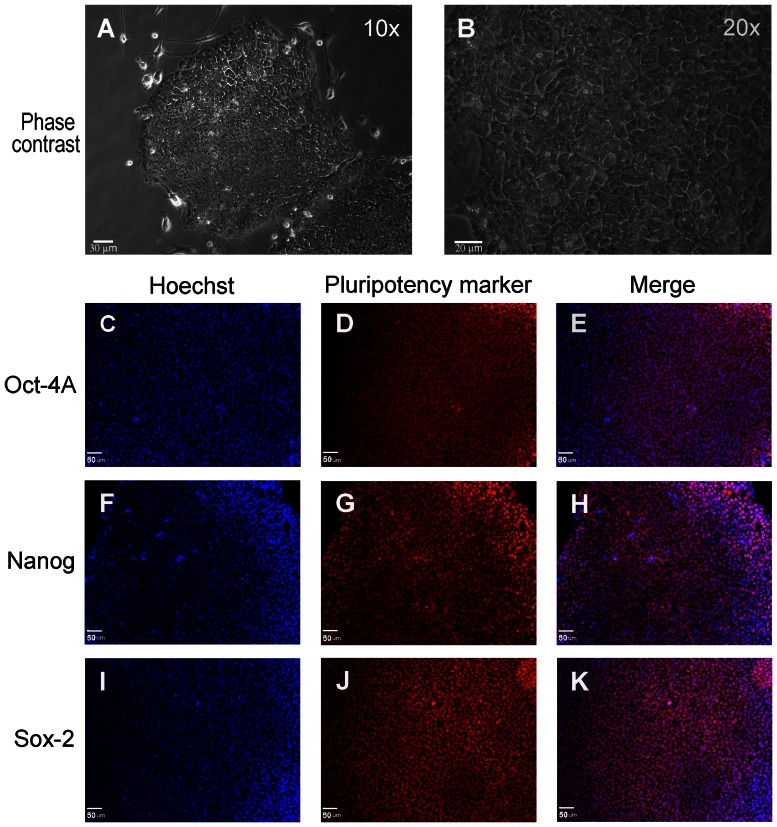
In vitro culture of vector-free hiPS cells in mTeSR1. (A, B) Vector-free hiPS cells cultured in mTeSR1 media on human ES qualified matrigel under serum- and feeder-free conditions, show a typical pluripotent cell morphology growing in colonies as monolayers. (B) is a magnified image (20x) of (A). (C to K) hiPS cells express pluripotency markers Oct-4A (C, D and E), Nanog (F, G and H) and Sox-2 (I, J and K). Nuclei were labeled with the nucleic acid counter-stain Hoechst-33342. Bar = 30 µm for A, 20 µm for B and 50 µm for C to K.

Human ES and iPS cells have typically been cultured on mitotically-inactivated MEFs that support their undifferentiated growth. However, stem cells cultured under these conditions start to express the non-human sialic acid residue Neu5Gc, which is immunogenic in humans [43]. Thus, a serum- and feeder-free condition is essential in human stem cell cultures for transplantation therapy. Under this condition, cells showed morphology typical of hES and hiPS cells growing in mono-layered colonies (Fig. 1A and 1B). These hiPS cells stained positive for pluripotency markers Oct4A (Fig. 1C, 1D, and 1E), Nanog (Fig. 1F, 1G, and 1H) and Sox-2 (Fig. 1I, 1J, and 1K). They were negative for SSEA-1, a marker for differentiating hiPS cells (data not shown). A full characterization of these hiPS cells can be found in a previous report [29] and on www.wicell.org.

### Neural Differentiation of hiPS Cells

**Figure 2:**
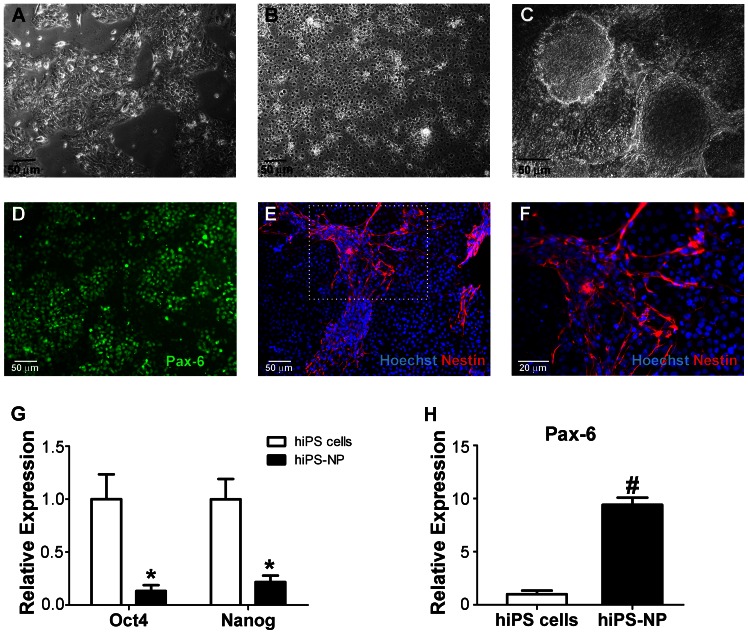
Differentiation of hiPS cells to Pax6- and Nestin-positive neural precursors. (A, B) After dissociation and plating on Matrigel, hiPS cells start to grow to confluence as monolayers. (A) shows hiPS cells 2 days after plating and (B) shows confluent hiPS cells 4 days after plating. (C–F) Eleven days after neural induction, hiPS cells form 3-dimensional neural rosette-like structures (C) that are immunoreactive to the neural precursor markers Pax-6 (D) and Nestin (E). (F) is a magnification of the area designated in (E). Green is Pax-6, blue is Hoechst-33342 and red is Nestin. Bar = 50 µm for A to E and 20 µm for F. (G and H) qRT-PCR analysis of pluripotency (G; Oct-4 and Nanog) and neural (H; Pax-6) markers showing a significant drop in Oct-4 and Nanog and significant increase in Pax-6 in hiPS-NPs compared to hiPS cells. GAPDH was used as internal control. Expression level in hiPS-NPs is showed relative to expression level in hiPS cells (n = 5 in all groups; *p<0.05, Two-way ANOVA with Bonferroni’s correction; #p<0.05, Student’s *t-test*).

Neural differentiation protocols used with human ES cells have traditionally been long and laborious processes [44], [45]. These protocols often depend on embryoid body formation or co-culture with other cell lines, as well as expensive recombinant factors such as Noggin [46]. We have characterized human ES cell neuronal differentiation and provided evidence that hiPS cells can be similarly differentiated to neurons using dorsomorphin and SB431542. Using this protocol, dissociated hiPS cells reached confluence in MEF-conditioned media (Fig. 2A and 2B). Following 11 days of neural induction, tri-dimensional rosette-like structures (Fig. 2C) and Pax6-positive neural precursors (Fig. 2D) were formed. After induction, we obtained 46±5% Pax6-positive cells (n = 5 independent assays). In addition, nestin, another marker of neuronal precursors, was positive in over 60% (n = 5) of the cells (Fig. 2E and 2F). Gene expression levels of key pluripotency genes (Oct-4 and Nanog) and the neural precursor marker (Pax-6) were also analyzed in hiPS cells and hiPS-NPCs using qRT-PCR. There was a significant drop in gene expression level of Oct-4 and Nanog after hiPS cell differentiation into hiPS-NPCs (Fig. 2G), concomitant with a significant increase in Pax-6 gene expression (Fig. 2H). These tests illustrated a loss of pluripotency phenotype and gain of neural characteristics by the end of the neural induction protocol.

### hiPS Cell-derived Neurons Exhibit Functional Neuronal Characteristics

**Figure 3:**
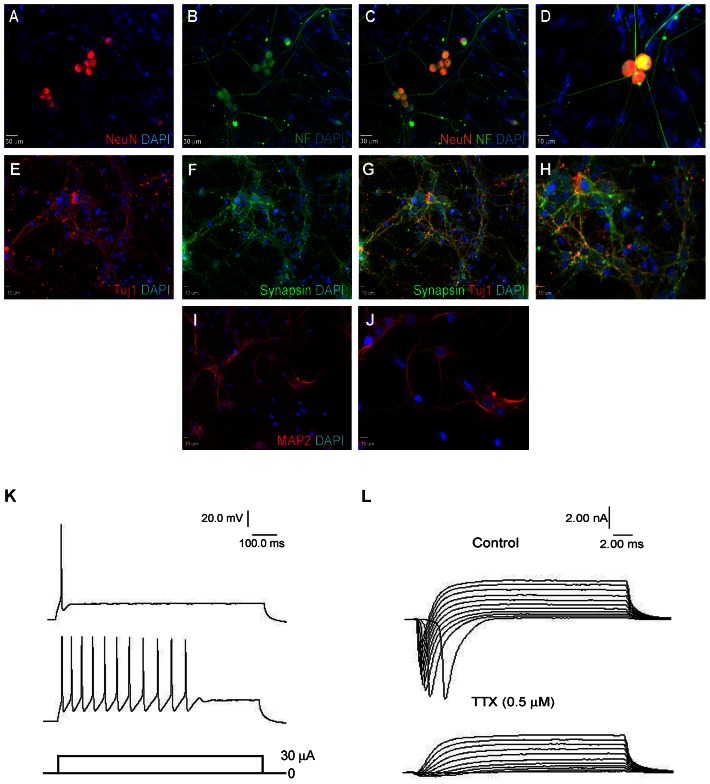
hiPS cell-derived neurons exhibit functional neuronal characteristics. (A–J) hiPS cells differentiate to mature neurons that express the neuronal markers NeuN (A), Neurofilament L (B), Tuj1 (E), Synapsin1 (F), and MAP2 (I) 4 weeks after terminal differentiation on Matrigel. (C) and (G) are merged images from (A–B) and (E–F), respectively. (D) is a magnified image of hiPS cell-derived neurons. (H) and (J) are magnifications of (G) and (I), respectively. Green is NF-L and synapsin; blue is DAPI; and red is NeuN, Tuj1 and MAP2. Bar = 30 µm for A to C, 10 µm for D–J. Images were chosen from areas dense with cells showing neuronal morphology. (K) Representative action potentials measured from hiPS cell-derived neurons 4 weeks after terminal differentiation. Most cells showed a single action potential spike while some exhibited a train of action potentials. The recorded action potentials are in response to 30 µA current injections under current clamp mode. n = 10 neurons. (L) Top panel shows rapid inward and slow outward currents elicited by step from −40 to 60 mV from a holding potential of −70 mV in hiPS cell-derived neurons 4 weeks after terminal differentiation. The rapid inward sodium current disappears with the addition of 0.5 µM TTX, a sodium channel blocker (lower panel). n = 10 neurons.

Four weeks after the 11-day neuronal induction protocol, hiPS cell-derived neurons formed extensive networks and stained positively for NeuN and Neurofilament L (Fig. 3A–3D), Tuj1 (β-tubulin III) and synapsin 1 (Fig. 3E–3H) and the mature neuronal marker MAP2 (Fig. 3I and 3J). NeuN was positive in 23±7% (n = 3) of all the Hoechst 33342-positive cells. Most NeuN-positive cells were additionally positive for Neurofilament. Over 80% of the NeuN-positive cells were also positive for Tuj1 and MAP2. Nestin fluorescent reactivity was negative in all cells.

Using whole-cell recording in electrophysiological assessments, the resting membrane potential of hiPS cell-derived neurons was 72.0±2.0 mV (n = 7). In current clamp recording mode, a depolarizing pulse of 1,000 ms duration triggered either a single action potential (70%) or repetitive spikes (30%) (Fig. 3K), suggesting the unique features of functional neurons (n = 10). TTX-sensitive sodium currents were elicited in all patched iPS cell-derived neurons, with average amplitude of 5.4±1.1 nA upon the depolarizing step from −70 mV to 0 mV (Fig. 3L).

### Transplantation of hiPS-NPCs after Focal Cerebral Ischemia in Mice

**Figure 4:**
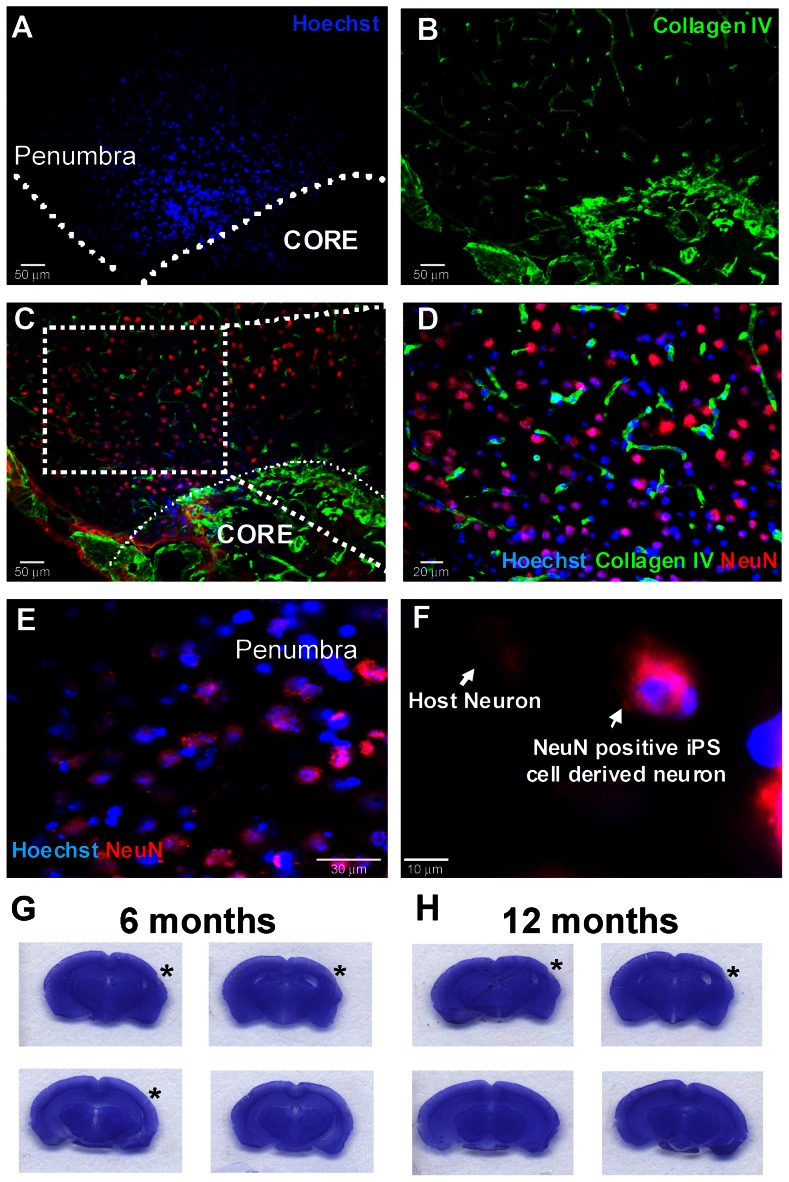
In vivo survival and differentiation of hiPS-NPs in the ischemic brain. (A–D) hiPS cells survive and differentiate to neurons 28 days after transplantation in the cortex penumbra region of stroke animals. The core region is delineated in (A) with the Hoechst 33342-positive hiPS cell-derived neurons residing in the penumbra. hiPS cell-derived neurons are identified by co-labeling Hoechst-33342 and NeuN. (D) is a magnified image of the marked area in (C). Blue is Hoechst-33342, green is Collagen IV (vessel marker) and red is NeuN. (E, F) are further magnifications to show the co-labeled Hoechst 33342-positive NeuN-positive hiPS cell-derived neurons. A Hoechst 33342-negative NeuN-positive host neuron is also shown in (F). Bar = 50 µm for A to C, 20 µm for D, 30 µm for E and 10 µm for F. (G–H) Nissl staining of brain sections representing brains of hiPS-NP transplanted animals 6 and 12 months after transplantation showing no indication of tumor formation. Asterisks indicate stroke location. N = 6 per group at each time point. (I) The *in vivo* experimental design for hiPS-NP transplantation and post-stroke experiments.

The effect of transplantation therapy using our hiPS-NPCs was tested in the focal ischemia barrel cortex stroke mouse model [34]. Cell transplantation into the ischemic cortex was performed 7 days after stroke. TUNEL staining was applied to detect cell death 48 hrs after transplantation. Approximately 15±3% of the Hoechst 33342-positive cells were positive for TUNEL staining (Figure S1C). *In vivo* neuronal differentiation was assessed by staining brain slices for NeuN 28 days after transplantation. The percentage of NeuN/Hoechst 33342 double positive cells was 26±5% among all Hoechst 33342-positive cells in the penumbra region (Fig. 4A–4F). Six and twelve months after transplantation, we inspected six animals (at each time point) for signs of tumor growth using Nissl staining. No signs of tumor or malignant growth could be identified at the site of transplantation in the penumbra, in the core, or in the surrounding regions (Fig. 4G and 4H). A timeline for the *in vivo* experiments is shown in Figure 4I.

### hiPS-NPC Transplantation Enhances Functional Behavioral Recovery after Stroke

While the focal ischemic stroke model mostly affects the barrel sensory cortical area, the ischemic core also extends into the sensorimotor cortex representing the upper limbs [42]. This makes the adhesive removal test a valuable tool in assessing sensorimotor recovery in our study.

**Figure 5:**
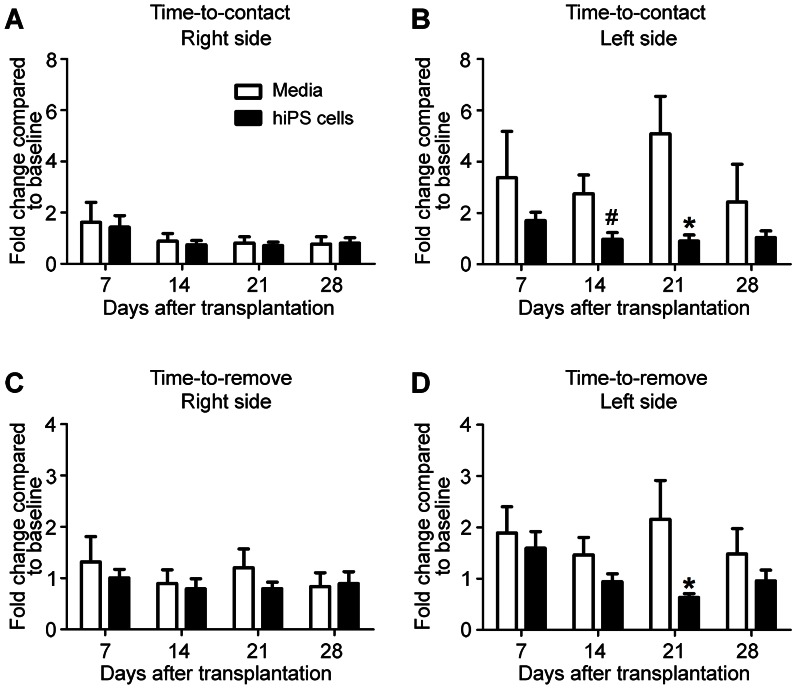
hiPS-NP transplantation enhances sensorimotor functional recovery. The adhesive-removal test was performed 7, 14, 21 and 28 days after transplantation. The values represent the ratio of the time-to-contact or time-to-remove the adhesive tape at each test date to the average time spent by the same animal immediately after training. (A and C) On the right side of the body, there was no difference in the time-to-contact or time-to-remove the adhesive tape between the media injection and cell transplantation groups and all values were centered on 1 indicating no difference before and after stroke induction. (B and D) On the affected (left) side, there was a significant difference in the time-to-contact (B) and time-to-remove (D) the adhesive tape between the media injection and cell transplantation groups at days 14 and 21, indicating a faster sensorimotor recovery with hiPS-NP transplantation. (n = 9 for both groups at all time points, *p<0.05, Two-way ANOVA with Bonferroni’s correction; #p<0.05, Student’s *t-test*).

The ischemic insult did not affect sensorimotor functions in the right paw corresponding to the contralateral cortex (Fig. 5A and 5C). On the other hand, left paws that associated with the ischemic cortex showed increased delays in time-to-contact and time-to-remove the adhesive tape in the sensorimotor test at all time points tested (Fig. 5B and 5D). Stroke mice in the cell transplanted group, however, maintained nearly unaltered time-to-contact and time-to-remove. In these mice, there was no significant difference between the performance of right and left paws. In comparison to stroke control group, mice transplanted with hiPS-NPCs performed significantly better at day 14 and 21. On day 14, there was a significant difference between the treated and stroke control groups in the time-to-contact measurement (p = 0.0364, n = 9). On day 21, the time-to-contact was 5.09±1.46 sec vs. 0.90±0.24 sec in the control and transplantation group, respectively, while time-to-remove was 2.16±0.75 sec vs. 0.63±0.07 sec (p<0.05 in both comparisons; n = 9 in each groups).

### Local Cerebral Blood Flow and Intrinsic Optical Signal Imaging

**Figure 6:**
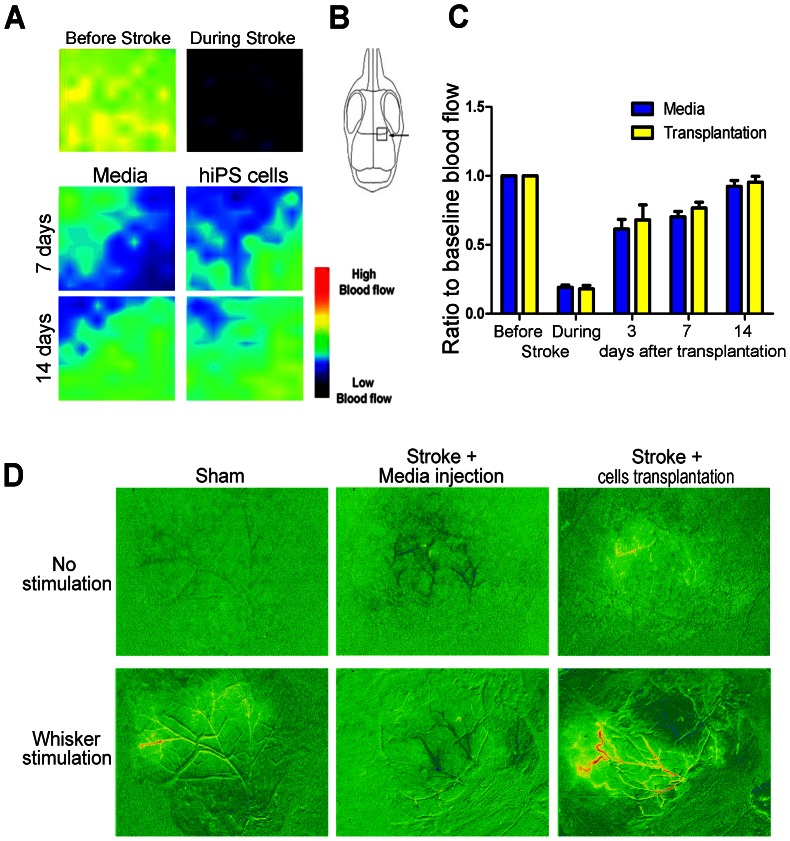
hiPS-NP transplantation restores neurovascular coupling after stroke. (A) Local cerebral blood flow is shown as pseudo-colored images of flow intensity before and after stroke and 3, 7 and 14 days post transplantation in the media or cell injected groups. A color scale for blood flow is shown in the lower right corner. (B) Blood flow was measured in areas around the medial border of the infarct (arrow). (C) Quantification of the mean intensity values normalized to before stroke mean values showing a slight increase in flow in the transplantation group without being statistically significant (n = 10 in each group; p<0.05; two-way ANOVA with Bonferroni’s post-hoc analysis). (D) Intrinsic optical signal imaging in control and transplantation animals 30 days after transplantation. Normal evoked barrel cortex responses are induced in sham animals after whisker stimulation. Stroke induction with only media injection resulted in the destruction of the neurovascular infrastructure and the disappearance of IOS in the barrel cortex after whisker stimulation. hiPS-NP transplantation after ischemic stroke restored the neurovascular architecture resulting in robust evoked responses after whisker stimulation. n = 5 in each group. Representative images are shown.

The area where imaging was analyzed is shown in Figure 6B. The recorded values are the mean values normalized to the blood flow before MCA occlusion for each mouse. After MCA ligation, local blood flow dropped to about 20% of the initial perfusion (Fig. 6A and 6C). At each time point after transplantation (3, 7 and 14 days), local cerebral blood flow was similar between the transplantation and media control group (n = 10 in each group; p>0.05).

To take a specific examination on the neural activity associated with local blood flow at the ischemic barrel cortex, intrinsic optical signals (IOS) evoked by whisker stimulation at the barrel cortex were assessed 30 days after transplantation. Functional imaging of IOS showed normal evoked barrel activity in sham animals after whisker stimulation. This evoked activity disappeared in stroke animals that received only media injection, indicating a damage to the whisker-barrel pathway and a defect in neurovascular coupling after barrel cortex stroke (Fig. 6D). hiPS-NPC transplantation noticeably enhanced the evoked response in the barrel cortex of stroke mice (Fig. 6D).

### Trophic Factor Expression in hiPS-NPCs before and after Transplantation

**Figure 7:**
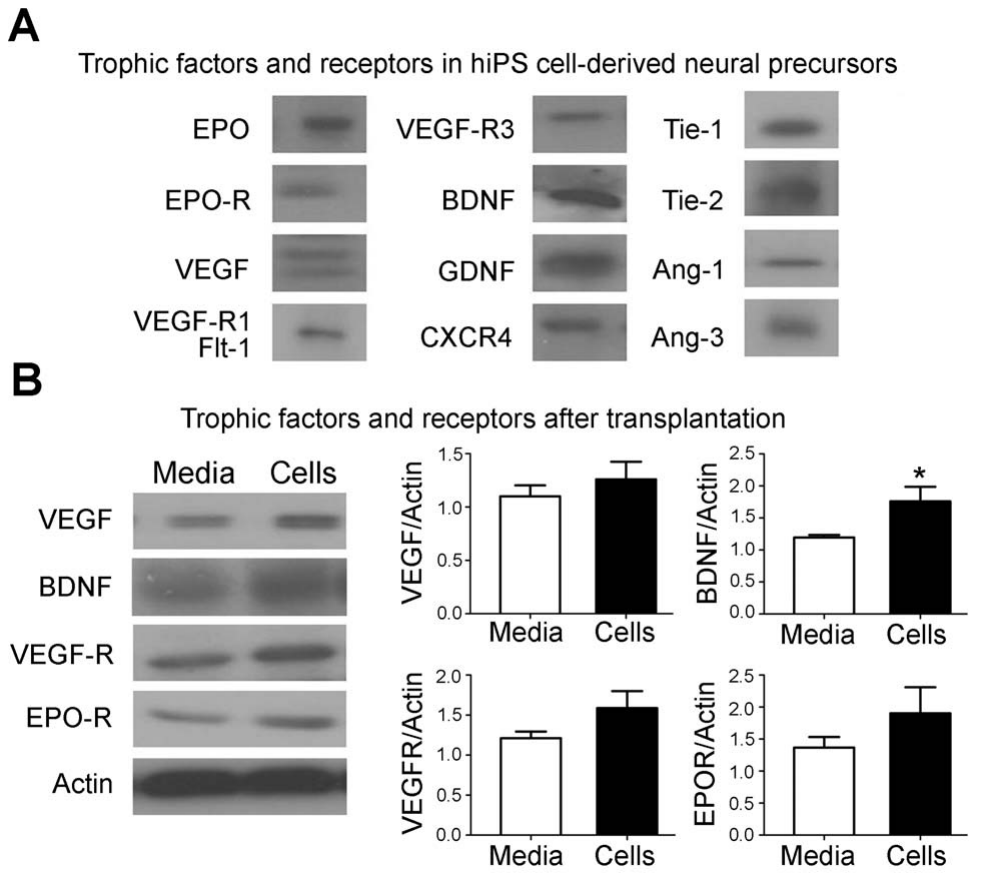
Trophic factor expression in hiPS-NPs before and after transplantation. (A) Qualitative assessment of trophic factors expression in hiPS-NPs. hiPS-NPs expressed EPO, EPO-R, VEGF, VEGF-R1 or Flt-1, VEGF-R3, BDNF, GDNF, CXCR4, Tie-1, Tie-2, Ang-1 and Ang-3. (B) BDNF expression level was significantly increased in the transplanted group compared to the media injected animals (*p<0.05; Student’s *t-test;* n = 6 for both groups). VEGF, VEGF-R and EPO-R were also increased but the difference was not significant between the two groups (p>0.05; Student’s *t-test*; n = 6 for both groups).

To understand the mechanisms of stem cell-induced benefits after transplantation in stroke animals, we measured the protein expression of some trophic factors and some of their receptors in hiPS-NPCs *in vitro* right before transplantation (Fig. 7A). Western blotting detected expression of EPO, EPO-R, VEGF, VEGF-R1 or Flt-1, VEGF-R3, BDNF, GDNF, CXCR4, Tie-1, Tie-2, Ang-1 and Ang-3 in these cells. The trophic factors were measured due to their important roles in cell survival, proliferation and migration and their ability to enhance endogenous repair mechanisms in the brain. We also compared expression of these factors in the penumbra of media-injection and cell treated stroke animals 14 days after transplantation (Fig. 7B). While most of the factors did not show difference between the two groups at this time point, the expression of VEGF, VEGF-R and EPO-R showed trends of increase, and BDNF expression significantly increased in the transplanted group compared to the media injected animals.

## Discussion

In this study, we investigated the therapeutic potential of vector-free and transgene-free hiPS-NPCs for the treatment of focal ischemic stroke in mice. *In vitro* experiments demonstrates that hiPS cells can be cultured in serum-free and feeder-free conditions in mTeSR1 and that these cells differentiate into neural precursors and can become fully mature neurons with typical electrophysiological properties. *In vivo* experiments show that, after transplantation, hiPS-NPCs survive and differentiate to neurons with mature neuronal markers. Behavioral tests demonstrate that hiPS-NPC transplantation enhances sensorimotor outcomes and restores functional networks within the whisker-barrel pathway in the barrel cortex stroke model. Western blot analysis showed that hiPS-NPCs express a variety of trophic factors and increase trophic support after transplantation. This report demonstrates the efficacy of vector-free hiPS cells in transplantation therapy that shows repair capability of these cells after ischemic stroke.

iPS cell derivation has substantially developed over the past 5 years. Lenti-viruses were first used to induce pluripotency to deliver a cocktail of transcription factors that included c-myc. c-Myc is a transcription factor involved in cell cycle regulation and proliferation and is aberrantly expressed in a variety of tumors [47]. Lenti-viral DNA can potentially integrate in the iPS cell genome in a random fashion which increases the risk of tumor formation and malignant transformation. A previous study showed that undifferentiated iPS cells could form more tumors in the ischemic compared to the intact brain [48]. Recent techniques using non-integrating episomal vectors circumvent the continuous presence of both lentiviral DNA and c-myc [29]. The present investigation provides the evidence that neural progenitors derived from vector-free and transgene sequence-free iPS cells are biologically safe (no abnormal cell growth) and effective in functional recovery after focal ischemic stroke.

Since their derivation, human ES and iPS cells have been routinely derived and cultured on MEFs [31], [49]. Accumulating evidence, however, shows that contamination of human pluripotent stem cells with animal products increases their immunogenicity due to the increased expression of a non-human sialic acid residue, Neu5Gc. Because of this concern and the fear of graft rejection with human transplantation studies, it is essential to use a culture system that is independent of feeders and of other animal products like bovine serum [30]. Acknowledging the fact that we used MEF-conditioned media in the differentiation process, our study shows the effectiveness of feeder- and serum-free maintenance conditions in experimental transplantation therapy utilizing hiPS cells.

While human ES and iPS cells have great potential in neuro-regenerative medicine, current protocols for their neural differentiation are highly heterogeneous with low yield of neuronal formation. This heterogeneity arises primarily from the inherent nature of the differentiation protocols. Whether using embryoid bodies (EBs) [44] or stromal supportive cells [50], [51], these protocols are non-reliable for human use because of the structural heterogeneity of the EBs and the continued presence of animal products with feeder cells. Given that, we utilized, with modifications, a recently developed adherent neural differentiation protocol avoiding EB formation and stromal cells [14]. This protocol relies on the dual inhibition of the SMAD pathway for efficient neural induction. While the original protocol relies on the use of Noggin and SB431542, we used a relatively inexpensive small molecule inhibitor of the bone morphogenic proteins (dorsomorphin) instead of the expensive Noggin. Neuronal differentiation of hiPS cells has also been shown to be less efficient and more variable when compared to hES cells [52]. Other reports however, have shown that the neural differentiation propensity of human pluripotent stem cells (ES or iPS cells) could be overcome using different protocols [14], [53]. In our work, we obtained Pax-6-positive neural precursors in 46% of the cells at the end of neural induction which is less than the 80% reported with the dual SMAD inhibition protocol [14]. Moreover, with RT-PCR, we only saw 3-fold increase in Pax-6 mRNA expression compared to the 10-folds reported earlier in the same study. In a previous study, we have shown that using the same protocol with H1 human ES cells, we got 80% pax-6- and 90% Nestin-positive cells (compare to 46% and 60%, respectively, with hiPS cells) [32]. These data indicate that while relatively efficient, the neural differentiation protocol of hiPS cells can still be optimized.

While previous studies have reported low survival of neural precursors after transplantation [54], [55], our high survival rate could be a reflection of a better *in vitro* culture (mTeSR) and differentiation (no EBs, no stromal cells) conditions. Moreover, control of transplantation parameters (timing, cell number, etc.) also increases survival. For example, hiPS-NPCs were transplanted 7 days after stroke when brain edema has subsided and acute excitotoxic factors such as glutamate accumulation and production of reactive oxygen species (ROS) are subsided. We recently demonstrated that hypoxic preconditioning before transplantation is a highly effective means of promoting survival and regenerative properties of transplanted cells [56]–[58]. It can be predicted that hypoxic preconditioning of virus-free hiPS cells would further enhance the survival and therapeutic efficacy of these cells. After local transplantation, cells appear in the core and ischemic penumbra without significant distribution to other brain areas. While we understand the complications of Hoechst-33342 labeling of the transplanted cells including interference with DNA replication and potential leakage into neighboring cells, Hoechst-33342 has been successfully used in other transplantation studies. It may be regarded as a limitation in this investigation that control animals received media injection but not cell controls. Previous investigations, however, have demonstrated that transplantation of differentiated cells such as cortical neurons does not shown similar therapeutic effects as seen with neural progenitor cells [5].

It is important to note that we did not use immunosuppressing drugs despite the immunocompatibility issue between the host (mouse) and the graft (human). In comparison tests, we did not observe any difference in the survival rates of human ES cell-derived neural precursors after transplantation into mice with and without administration of an immunosuppressant. Moreover, we did not see immune rejection in our transplantation experiments using human iPS-NPCs in the mouse stroke model. The underlying mechanism of this immunological tolerance is not clear. It is well-known, however, that some widely used stem cells such as bone marrow mesenchymal stem cells (BMSCs) have immunomodulatory function after transplantation. The general effects are thought to ‘skew’ the host immune response toward anti-inflammatory/tolerant phenotypes [38]. It is possible that iPS cells and/or iPS-NPCs have the similar immunomodulatory action in the host tissue, this putative mechanism of function of these cells needs to be specifically elucidated. It is clinically important to point out that immune suppression drug treatment can be detrimental to endogenous healing, as some immune responses are actually neuroprotective and necessary to endogenous neurogenesis [59]–[62]. Immune suppression also may reduce the inflammatory signals that attract stem cells to the site of injury [63] or increase the risk of tumor formation [64], [65]. Given these issues and the health problems that can be caused by long-term immune suppression [66], it is clear that the immunomodulatory function of transplanted cells is an obvious advantage in clinical treatments and the use of immunosuppressants should be avoided whenever possible.

Several studies have explored the use of iPS cells in various models of stroke [38], [67]–[71]. All of these studies, however, used viruses in the process of iPS cell induction and one of them did not show any functional benefit after transplantation. In conjunction with several reports about stem cell transplantation in stroke models [5], [72], [73], we have seen improved behavioral performance in the adhesive removal test after neural precursor transplantation. Although neurovascular plasticity usually results in some degree of functional recovery after stroke, transplantation of hiPS-NPCs significantly accelerated the rate of recovery. Moreover, IOS imaging, an indicator of neurovascular coupling, showed significantly better neurovascular architecture and functional activity after stroke in mice received hiPS-NPC transplantation. We also detected expression of trophic factors in hiPS-NPCs before transplantation and an increase in trophic support especially BDNF after transplantation, indicating that the transplanted cells could provide trophic support to the ischemic brain. BDNF has been associated with neural stem cell-induced benefits in transgenic model of Alzheimer disease [74] and has a critical role in recovery after ischemic stroke [75], [76]. Another imperative mechanism of stem cell therapy is possible integration of differentiated cells in brain circuits. We showed recently that although increased trophic support is an important benefit of transplantation therapy using BMSCs, the possibility of cell replacement achieved by neurally differentiated cells should not be excluded especially when cell survival, migration, and differentiation issues can be adequately addressed [77]. Being pluripotent stem cells, iPS cells are inherently suitable for cell replacement therapies. This tissue repair mechanism with virus-free, transgene-free human iPS cells deserves further investigations.

## Supporting Information

Figure S1 **Experimental design and stroke model.** (A) During stroke induction, blood flow drops to less than 20% compared to that before MCA and CCA occlusion (n = 6, *p<0.05, Student’s *t-test*). (B) Focal ischemic barrel cortex stroke as shown by the TTC stain. The stroke core (dotted area) and the ischemic penumbra (filled area) are marked and represent the areas of cell transplantation. (C) TUNEL staining of the transplanted hiPS-NPs indicating that most cells survive 2 days after transplantation. Green is TUNEL and blue is Hoechst-33342. Bar = 30 µm for D.
(TIF)
