# Supplementary figures and images for: Vector-Free and Transgene-Free Human iPS Cells Differentiate into Functional Neurons and Enhance Functional Recovery after Ischemic Stroke in Mice
Source: PLoS One. 2013 May 23;8(5):e64160. doi: 10.1371/journal.pone.0064160 (PMC3662762; doi:10.1371/journal.pone.0064160)

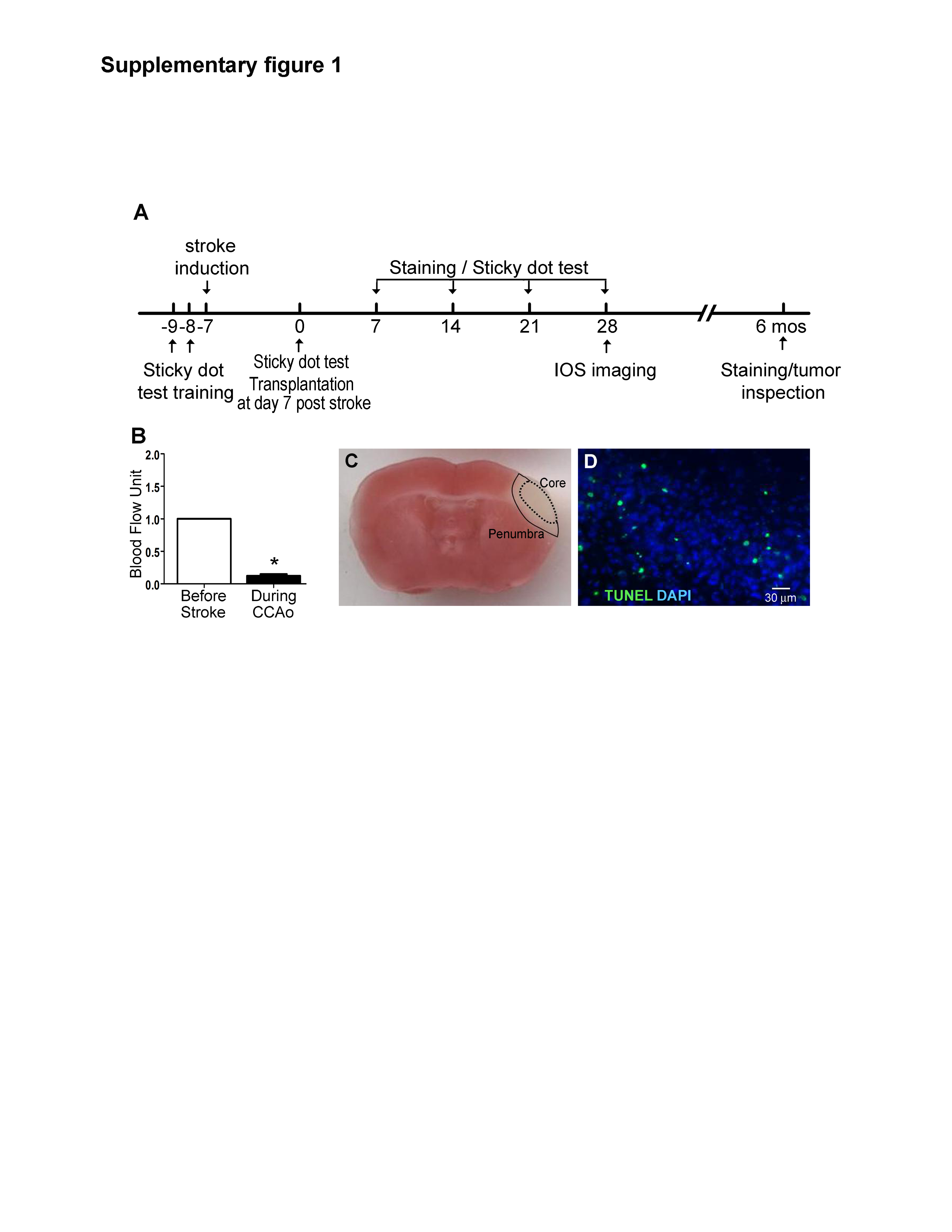

Supplement: Figure S1 — Experimental design and stroke model. (A) During stroke induction, blood flow drops to less than 20% compared to that before MCA and CCA occlusion (n = 6, *p<0.05, Student’s t-test). (B) Focal ischemic barrel cortex stroke as shown by the TTC stain. The stroke core (dotted area) and the ischemic penumbra (filled area) are marked and represent the areas of cell transplantation. (C) TUNEL staining of the transplanted hiPS-NPs indicating that most cells survive 2 days after transplantation. Green is TUNEL and blue is Hoechst-33342. Bar = 30 µm for D. (TIF) [file pone.0064160.s001.tif]
